# Exploring the Joint Impact of METS‐VF and Functional Limitation on Cardiometabolic Multimorbidity Risk

**DOI:** 10.1155/jdr/6450867

**Published:** 2026-06-12

**Authors:** Xuhui Chen, Ying Wang, Jiaofen Wu, Yulian He, Huihui Chen, Honghua Ye, Jianhui Liu

**Affiliations:** ^1^ Department of Pharmacy, Ningbo Medical Center Lihuili Hospital, Ningbo, Zhejiang, China, nbws.gov.cn; ^2^ Department of Cardiology, Ningbo Medical Center Lihuili Hospital, Ningbo, Zhejiang, China, nbws.gov.cn

**Keywords:** cardiometabolic multimorbidity, CHARLS, cohort study, functional limitation, metabolic score for visceral fat (METS-VF), visceral obesity

## Abstract

**Background:**

Cardiometabolic multimorbidity (CMM) has emerged as a primary public health challenge, significantly increasing mortality risk and reducing quality of life. Although the metabolic score for visceral fat (METS‐VF) and functional limitation are recognized individually as risk factors for cardiometabolic health, the bidirectional relationship between functional limitation and CMM suggests complex underlying mechanisms. However, research investigating the independent and joint effects of METS‐VF (representing visceral obesity) and functional limitation on the risk of CMM onset is lacking. This study is aimed at systematically elucidating these associations to provide evidence for targeted interventions.

**Methods:**

This prospective cohort study utilized data from the China Health and Retirement Longitudinal Study (CHARLS). A total of 8140 participants were included in the cross‐sectional analysis, and a sub‐cohort of 5356 individuals free of CMM at baseline was followed longitudinally. CMM was defined as the coexistence of at least two cardiometabolic diseases: heart disease, diabetes, stroke, or hypertension. Functional limitation was assessed using activities of daily living (ADL) and instrumental ADL (IADL) scales. Multivariable Cox proportional hazards regression and restricted cubic spline (RCS) analysis were employed to evaluate the association between METS‐VF, functional limitations, and CMM incidence.

**Results:**

During the follow‐up period, METS‐VF demonstrated a nonlinear positive dose‐response relationship with CMM risk. In the longitudinal analysis, participants in the high METS‐VF group (defined by the RCS inflection point) exhibited a significantly elevated risk of developing CMM compared to the low‐risk group (HR = 1.77, 95% CI: 1.39–2.26) after adjusting for confounders. Continuous functional limitations were also independently associated with incident CMM (HR = 1.12, 95% CI: 1.08–1.16). Notably, a joint effect was observed; participants with both high METS‐VF and functional limitations faced the highest risk (HR = 2.43, 95% CI: 1.78–3.31). Receiver operating characteristic (ROC) analysis indicated that the combined model incorporating both factors achieved superior predictive accuracy (AUC = 0.680) compared to single‐factor models.

**Conclusion:**

METS‐VF and functional limitations are strong independent predictors of CMM, and their simultaneous presence exerts a significant joint effect on disease risk. The integration of METS‐VF and functional assessment into clinical practice can enhance CMM risk stratification and facilitate the early identification of vulnerable high‐risk subgroups.

## 1. Introduction

Amidst global population aging and lifestyle transitions, cardiometabolic diseases (CMDs) have emerged as a primary public health challenge [1]. In clinical practice, multiple cardiometabolic conditions, such as diabetes, heart disease, and stroke, frequently coexist within the same individual, constituting a complex health status known as “cardiometabolic multimorbidity (CMM)” [2, 3]. CMM is not merely a simple aggregation of diseases; its health detriment far surpasses the sum of individual conditions, significantly increasing all‐cause mortality risk, reducing life expectancy, and severely impacting patients′ quality of life and mental health [4]. Consequently, the precise identification and effective intervention of high‐risk factors for CMM are of paramount importance for delaying disease progression and promoting healthy aging.

Among the myriad risk factors, the excessive accumulation of visceral fat is considered a core pathophysiological link contributing to the development of CMM [5]. As a metabolically active tissue, dysfunctional visceral adipose tissue (VAT) systemically impairs cardiovascular and metabolic health by releasing pro‐inflammatory cytokines and exacerbating insulin resistance [6]. However, traditional obesity assessment metrics like body mass index (BMI), which have long been widely used, are increasingly recognized for their limitations in accurately assessing cardiometabolic risk due to their inability to effectively reflect body fat distribution [7]. To address this, novel biomarkers have been developed, among which the metabolic score for visceral fat (METS‐VF) has shown significant potential [8]. This score integrates readily available anthropometric, metabolic, and demographic data to construct a comprehensive index that efficiently reflects visceral fat content and its functional state. A growing body of evidence confirms that the METS‐VF index demonstrates superior predictive performance over traditional anthropometric and metabolic indicators in identifying individuals at high risk for CMDs, including Type 2 diabetes and hypertension [9, 10].

Concurrently, functional limitation—characterized by limitations in performing basic activities of daily living (ADL) or more complex instrumental ADL (IADL)—presents another severe health challenge during the aging process [11]. Extensive research indicates that functional limitation is closely associated with falls, cognitive decline, and increased mortality risk [12, 13]. Research has increasingly focused on the complex bidirectional relationship between functional limitation and cardiometabolic health. Substantial evidence confirms CMM as a significant predictor of functional limitation development [14]. Furthermore, recent evidence from longitudinal studies suggests this relationship may be reciprocal—functional limitation itself may conversely increase an individual′s risk of developing CMDs [15]. Potential mechanisms may involve reduced physical activity levels resulting from functional limitations, as well as consequent negative psychological emotions, both of which are recognized risk factors for CMDs [16, 17].

Although existing evidence has separately revealed the association between METS‐VF and single CMDs, it has also revealed a bidirectional link between functional limitation and CMM, there is currently a lack of research combining these two critical risk factors—METS‐VF (representing visceral obesity) and functional limitation (representing physical function)—to jointly explore their impact on the risk of CMM onset. Given that METS‐VF comprehensively assesses visceral fat dysfunction whereas functional limitation reflects overall health status and lifestyle limitations, investigating their independent and joint effect holds significant theoretical and practical value for constructing more comprehensive CMM risk prediction models, precisely pinpointing vulnerable subgroups, and designing targeted multidomain intervention strategies. Therefore, this study is aimed at systematically elucidating the association of METS‐VF and functional limitation with the risk of CMM, providing new scientific evidence for clinical prevention and treatment.

## 2. Methods

### 2.1. Study Population

This study utilized data from the China Health and Retirement Longitudinal Study (CHARLS), a nationally representative prospective cohort that systematically tracks multidimensional indicators among Chinese adults aged 45 years and older. The study collects comprehensive data spanning sociodemographic profiles, economic status, health conditions, physical functioning, and biomarker measurements through standardized protocols. The national baseline survey of CHARLS was launched in 2011, recruiting 17,708 participants, with follow‐up surveys conducted every 2 years thereafter. The CHARLS research protocol received ethical approval from the Biomedical Ethics Review Committee of Peking University (Approval No. IRB00001052‐11015). All study procedures were conducted in accordance with the ethical principles of the Declaration of Helsinki, and written informed consent was obtained from all participants prior to their enrollment in the study. Public data for this study are available from the official website (http://charls.pku.edu.cn/).

**Figure 1:**
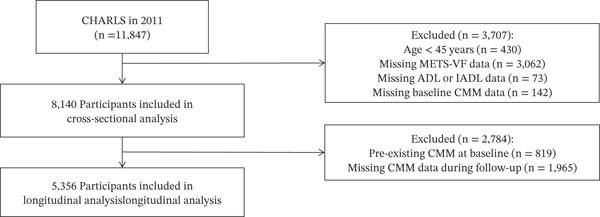
Flow chart of sample selection and the exclusion criteria.

This study utilized data from the CHARLS, initiated with the 2011 baseline survey (Wave 1) and incorporating follow‐up data from Waves 2 through 4, extending through 2018. To ensure analysis accuracy and longitudinal integrity, we established the following exclusion criteria: (1) Participants presenting with CMM at baseline; (2) individuals with missing data on outcome events (CMM) or key exposure variables (e.g., indices required for METS‐VF calculation, functional limitation assessment) at baseline or during follow‐up; (3) individuals completely lost to follow‐up in subsequent surveys. After applying these exclusions, the final analytical sample for the cross‐sectional study comprised 8140 participants, from which a longitudinal cohort of 5356 individuals without CMM was followed for incident events. The complete participant selection process is detailed in Figure 1.

### 2.2. Assessment of CMM Events

CMM was characterized by the coexistence of at least two of the following CMDs: heart disease, diabetes, stroke, and hypertension [18]. Cases of heart disease and stroke were established primarily through self‐reported history of physician‐diagnosed disease. During each follow‐up, data collection for heart disease and stroke relied on standardized questions in which participants were asked if they had ever received a physician′s diagnosis for these conditions (with heart disease explicitly including myocardial infarction, coronary heart disease, angina, congestive heart failure, and other related conditions). Additionally, current use of any cardiovascular‐related medication was used as an auxiliary criterion for identifying these diseases [19]. The diagnosis of diabetes employed a comprehensive approach using multiple information sources to improve accuracy. Participants were identified as having diabetes if they met any of the following conditions: (1) Self‐reported diagnosis of diabetes or hyperglycemia by a doctor; (2) fasting blood glucose (FBG) level ≥ 7.0 mmol/L; (3) glycated hemoglobin (HbA1c) level ≥ 6.5%; (4) current use of any glucose‐lowering medication [20]. Hypertension was identified by either of the following: (1) Evidence of clinical diagnosis (based on self‐reported physician diagnosis or current antihypertensive medication use); (2) objective measurement (SBP ≥ 140 mmHg or DBP ≥ 90 mmHg) [21]. In the longitudinal analysis of this study, we included participants free of CMM at baseline, with the follow‐up starting from the completion date of the 2011 baseline survey. The time of the outcome event was defined as the follow‐up year in which the participant first reported meeting the CMM diagnostic criteria. For participants who did not develop CMM during the entire follow‐up period, follow‐up duration was defined as the time interval from their baseline assessment to the final completed follow‐up survey.

### 2.3. Assessment of METS‐VF

METS‐IR and METS‐VF were calculated as follows:

• METS‐IR = [ln((2 × FPG) + TG) × BMI]/[ln (HDL‐C)] [22].
• METS‐VF = 4.466+0.011 × (ln(METS‐IR))^3^ + 3.239 × (ln(WHtR))^3^ + 0.319 × sex + 0.594 × ln (age) [8].

To strictly establish the temporal sequence between exposure and outcome, the METS‐VF score for each participant was calculated utilizing biomarker data exclusively collected during the 2011 national baseline survey (Wave 1). In these baseline measurements, TG represents serum triglycerides, FPG represents fasting plasma glucose, and HDL‐C represents high‐density lipoprotein cholesterol. All hematological data were measured in a fasting state. BMI was calculated as weight in kilograms divided by the square of height in meters (kg/m^2^). The waist‐to‐height ratio (WHtR) was calculated as waist circumference (cm) divided by standing height (cm). Additionally, sex was categorized as a binary variable (female, 0; male, 1), and age was coded in years.

### 2.4. Functional Limitations

This study utilized two dimensions—ADL and IADL—to comprehensively assess participants′ functional limitation status. ADL assessment was based on the classic Katz Index [23], covering six self‐care activities essential for maintaining basic independent living: eating, bathing, dressing, transferring (getting in and out of bed), toileting, and controlling urination/defecation. IADL assessment was derived from the scale proposed by Lawton and Brody [24], containing five instrumental activities essential for independent community living: managing finances, taking medications, shopping for groceries, performing housework, and preparing hot meals. In the CHARLS survey, for each activity mentioned above, participants were asked if they experienced difficulty performing it. Options included “No difficulty,” “Have difficulty but can still complete it,” “Have difficulty and need help,” and “Unable to complete it.” Following standard criteria from previous studies, participants answering “Have difficulty and need help” or “Unable to complete it” for any ADL or IADL activity were defined as having ADL disability or IADL disability, respectively [25]. In this study, participants presenting with either ADL disability or IADL disability were considered to have functional impairment (functional limitations).

### 2.5. Covariates

A comprehensive set of potential confounding factors was collected at baseline through standardized procedures. Detailed sociodemographic and lifestyle information was obtained via face‐to‐face structured questionnaires, including age, sex, marital status (married, single), educational attainment (categorized as illiterate, primary school, middle school, and high school or above), smoking status (current smoker, exsmoker, nonsmoker), and drinking status (drink more than once a month, drink but less than once a month, nondrinker). Clinical and anthropometric indices were measured by professionally trained staff, including height and weight for BMI calculation (kg/m^2^). Biochemical indicators were detected from venous blood samples collected after at least 8 h of fasting; samples were sent to a central laboratory for analysis, covering FBG, HbA1c, serum creatinine (Cr), and a complete lipid profile. Furthermore, to stringently control for clinical confounding, an extensive panel of baseline comorbidities was assessed, encompassing hypertension, diabetes, heart disease, stroke, cancer, lung disease, psychiatric problems, arthritis, liver disease, kidney disease, stomach/digestive diseases, asthma, and memory problems. Baseline medication use was also included as a covariate, specifically western and traditional Chinese antihypertensives, oral antidiabetics, insulin, and psychiatric medications.

### 2.6. Statistical Analysis

Continuous variables were initially assessed for normality. Normally distributed continuous variables are expressed as mean ± standard deviation (SD) and were compared using Student′s *t*‐test. Skewed continuous variables are presented as medians with interquartile ranges (IQR) and were compared using the Mann–Whitney *U* test. Categorical variables are presented as frequencies and percentages, with differences across groups evaluated using chi‐square or Fisher′s exact tests. In the cross‐sectional analysis, multivariable logistic regression was utilized to estimate the association between METS‐VF and CMM. In the longitudinal analysis, Kaplan–Meier curves were plotted to estimate the cumulative incidence of CMM, with differences evaluated by the log‐rank test. Cox proportional hazards regression models were subsequently employed to calculate hazard ratios (HRs) and 95% confidence intervals (CIs) for incident CMM. To rigorously account for potential confounders, three models were constructed: Model 1 was unadjusted; Model 2 was adjusted for age, sex, education level, marital status, smoking, and drinking status; Model 3 (the fully adjusted model) further incorporated Cr and comprehensive baseline medication use (western/traditional Chinese antihypertensive medications, oral antidiabetic drugs, insulin, and psychiatric medications), and baseline comorbidities. To optimize confounding control and preclude mathematical collinearity, the inclusion of baseline comorbidity covariates varied between the cross‐sectional and longitudinal analyses. For the cross‐sectional analysis, Model 3 adjusted for nine noncardiometabolic chronic conditions (cancer, lung disease, psychiatric problems, arthritis, liver disease, kidney disease, stomach/digestive diseases, asthma, and memory problems). For the longitudinal cohort analysis, to rigorously account for the baseline risk profile, Model 3 additionally adjusted for the four baseline individual components of CMM (hypertension, diabetes, heart disease, and stroke), resulting in a comprehensive adjustment of 13 baseline comorbidities. To explore the potential nonlinear dose‐response relationship between continuous METS‐VF and the risk of CMM, restricted cubic spline (RCS) modeling with four knots was performed using the “rms” package. To establish an objective and biologically plausible cutoff value, piecewise linear regression was subsequently applied using the “segmented” package in R. This algorithm mathematically identified a structural break (inflection point) in the baseline data. Based on this empirically derived inflection point, participants were categorized into “Low METS‐VF” and “High METS‐VF” groups. Furthermore, functional limitation was dichotomized into the presence (score > 0) versus the absence (score = 0) of functional impairment. Accordingly, to comprehensively evaluate their combined impact, participants were stratified into four joint risk categories: “Neither high,” “ Functional limitation only,” “High METS‐VF only,” and “Both high”. The joint impact of these groups on CMM incidence was assessed using Cox models, with the “Neither high” group serving as the reference. Subgroup analyses were performed to evaluate potential heterogeneity across strata of demographic and lifestyle factors. To ensure the exceptional robustness of our findings, three stringent sensitivity analyses were conducted based on Model 3: (1) Excluding all participants receiving baseline medications to eliminate pharmacological confounding; (2) performing a lag analysis by excluding participants who developed CMM within the first 2 years of follow‐up to minimize reverse causation; (3) restricting the analysis to a “super‐clean” subcohort by completely excluding individuals with any single baseline component of CMM. Receiver operating characteristic (ROC) curve analysis was used to assess predictive values, and differences in the areas under the curves (AUCs) were compared using the DeLong test. Finally, to explore the potential pathophysiological mechanisms linking METS‐VF to incident CMM, a formal mediation analysis was performed to quantify the mediating role of systemic inflammation. Baseline C‐reactive protein (CRP) levels were log‐transformed to approximate a normal distribution. Using the mediation package in R, we employed 500 bootstrap resamples to calculate the average causal mediation effect (ACME), average direct effect (ADE), and the proportion of the total effect mediated by CRP. All statistical analyses were performed using R Version 4.3.2. A two‐sided *p* value < 0.05 was considered statistically significant.

## 3. Results

### 3.1. Characteristics of Participants in the Cross‐Sectional Study

The baseline cross‐sectional analysis included a total of 8140 participants. Based on the nonlinear relationship identified via RCS analysis (detailed in the “Dose‐Response” section), participants were dichotomized into a “Low METS‐VF” group (METS − VF < 6.17, *n* = 1400) and a “High METS‐VF” group (METS − VF ≥ 6.17, *n* = 6740) according to the derived inflection point. Baseline characteristics stratified by METS‐VF status are presented in Table 1. Participants in the high METS‐VF group were generally older (59.82 ± 9.32 vs. 57.20 ± 9.13 years, *p* < 0.001) and exhibited significantly poorer anthropometric and biochemical profiles, including higher BMI, waist circumference, FBG, HbA1c, triglycerides, and CRP levels (all *p* < 0.001). Furthermore, the high METS‐VF group demonstrated a significantly higher CMD burden, with elevated prevalences of hypertension (46.9% vs. 24.8%), diabetes (18.2% vs. 7.6%), and heart disease (12.9% vs. 8.3%) (all *p* < 0.001). Consistently, corresponding medication use was markedly more frequent in the high METS‐VF group. Significant differences were also observed in marital status, and smoking status between the two groups (all *p* < 0.05). Ultimately, the prevalence of preexisting CMM at baseline was substantially higher among individuals with high METS‐VF compared to those with low METS‐VF (11.4% vs. 3.7%, *p* < 0.001).

**Table 1:** Baseline characteristics of participants stratified by METS‐VF.

Variable	Overall (*N* = 8140)	Low METS‐VF (*N* = 1400)	High METS‐VF (*N* = 6740)	*p*
Age, years, mean (SD)	59.37 (9.34)	57.20 (9.13)	59.82 (9.32)	< 0.001
Sex, n (%)				0.054
Female	4350 (53.4)	715 (51.1)	3635 (53.9)	
Male	3790 (46.6)	685 (48.9)	3105 (46.1)	
Educational level, *n* (%)				0.332
Illiterate	2426 (29.8)	417 (29.8)	2009 (29.8)	
Primary school	3330 (40.9)	548 (39.1)	2782 (41.3)	
Middle school	1580 (19.4)	293 (20.9)	1287 (19.1)	
High school and above	804 (9.9)	142 (10.1)	662 (9.8)	
Marital status, *n* (%)				0.013
Married	6102 (84.0)	1076 (86.4)	5026 (83.6)	
Single	1158 (16.0)	169 (13.6)	989 (16.4)	
Smoking status, *n* (%)				< 0.001
Current smoker	1917 (26.5)	398 (32.0)	1519 (25.3)	
Exsmoker	1265 (17.5)	186 (15.0)	1079 (18.0)	
Nonsmoker	4062 (56.1)	659 (53.0)	3403 (56.7)	
Drinking status, *n* (%)				0.216
Nondrinker	4888 (67.7)	814 (65.7)	4074 (68.1)	
Drink but < once a month	565 (7.8)	108 (8.7)	457 (7.6)	
Drink ≥ once a month	1772 (24.5)	317 (25.6)	1455 (24.3)	
METS‐VF, mean (SD)	6.67 (0.63)	5.78 (0.39)	6.86 (0.50)	< 0.001
Waist circumference, cm, mean (SD)	85.38 (10.14)	72.25 (4.77)	88.11 (8.73)	< 0.001
BMI, kg/m^2^, median [IQR]	23.15 [20.82, 25.82]	19.62 [18.29, 20.91]	23.93 [21.82, 26.38]	< 0.001
Fasting blood glucose, mean (SD)	110.05 (35.64)	101.87 (22.01)	111.75 (37.63)	< 0.001
HbA1c, %, mean (SD)	5.29 (0.82)	5.12 (0.56)	5.33 (0.86)	< 0.001
Triglycerides, median [IQR]	104.43 [74.34, 150.45]	80.54 [62.83, 112.39]	109.74 [78.76, 160.18]	< 0.001
LDL‐C, mean (SD)	117.74 (34.94)	112.04 (32.41)	118.93 (35.33)	< 0.001
HDL‐C, mean (SD)	51.41 (15.36)	58.65 (16.01)	49.90 (14.78)	< 0.001
Total cholesterol, mean (SD)	194.78 (38.87)	187.98 (36.95)	196.19 (39.11)	< 0.001
Serum creatinine, mean (SD)	0.81 (0.27)	0.80 (0.20)	0.81 (0.28)	0.128
CRP, median [IQR]	1.05 [0.56, 2.19]	0.72 [0.41, 1.53]	1.12 [0.60, 2.30]	< 0.001
Hypertension	3507 (43.1)	347 (24.8)	3160 (46.9)	< 0.001
Diabetes	1333 (16.4)	106 (7.6)	1227 (18.2)	< 0.001
Heart disease	984 (12.1)	116 (8.3)	868 (12.9)	< 0.001
Stroke	215 (2.6)	26 (1.9)	189 (2.8)	0.055
Kidney disease	476 (5.9)	89 (6.4)	387 (5.8)	0.390
Antihypertensive drug (western)	1577 (19.4)	116 (8.3)	1461 (21.7)	< 0.001
Antihypertensive drug (Chinese)	208 (2.6)	11 (0.8)	197 (2.9)	< 0.001
Antidiabetic drug (western)	286 (3.5)	15 (1.1)	271 (4.0)	< 0.001
Antidiabetic drug (Chinese)	63 (0.8)	3 (0.2)	60 (0.9)	0.014
Insulin therapy	55 (0.7)	0 (0.0)	55 (0.8)	0.001
Psychiatric drug	47 (0.6)	8 (0.6)	39 (0.6)	1.000
Functional limitation score, median [IQR]	0.00 [0.00, 1.00]	0.00 [0.00, 1.00]	0.00 [0.00, 1.00]	< 0.001
Preexisting CMM at baseline, *n* (%)	819 (10.1)	52 (3.7)	767 (11.4)	< 0.001

### 3.2. Survival Analysis of Different Risk Groups

**Figure 2:**
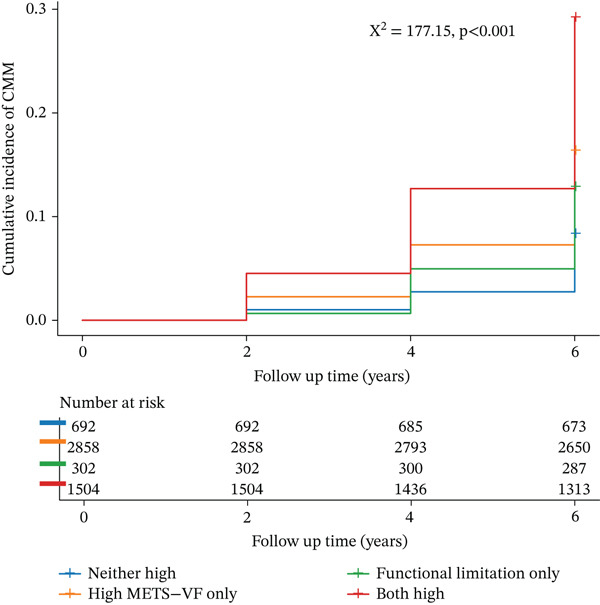
Kaplan—Meier curve of cumulative incidence of incident CMM based on METS‐VF and functional limitation status.

The cumulative incidence of CMM was evaluated using Kaplan–Meier analysis. As shown in Figure 2, the four joint risk categories (stratified by METS‐VF and functional limitation status) demonstrated significant divergence in incident CMM risk (log‐rank test, *χ* ^2^ = 177.15, *p* < 0.001). Specifically, the cumulative incidence of CMM was lowest in the “Neither high” group, followed progressively by the “Functional limitation only” group and the “High METS‐VF only” group, ultimately peaking in the “Both high” group. Over the 6‐year follow‐up period, the “Both high” group consistently experienced the steepest and most dramatic increase in cumulative incidence.

### 3.3. Association of METS‐VF and Functional Limitations With CMM in Cross‐Sectional Analysis

The cross‐sectional analysis revealed significant associations between METS‐VF, functional limitations, and the prevalence of CMM (Table 2). Based on the RCS‐derived inflection point, participants in the high METS‐VF group demonstrated substantially greater odds of prevalent CMM compared to the low METS‐VF reference group in the fully adjusted model (Model 3) (OR = 3.37, 95% CI: 2.36–4.96, *p* < 0.001). When analyzed as a continuous variable, each 1‐unit increase in METS‐VF was independently associated with higher odds of CMM (OR = 2.54, 95% CI: 2.09–3.12, *p* < 0.001). Similarly, functional limitation was positively associated with prevalent CMM (OR = 1.13 per 1‐unit increase, 95% CI: 1.08–1.17, *p* < 0.001).

**Table 2:** Association between METS‐VF Groups and CMM in cross‐sectional analyses.

Variable	Model 1 OR (95% CI)	Model 1 *p*	Model 2 OR (95% CI)	Model 2 *p*	Model 3 OR (95% CI)	Model 3 *p*
METS‐VF (Continuous, per 1 unit)	3.06 (2.61–3.61)	< 0.001	2.61 (2.20–3.12)	< 0.001	2.54 (2.09–3.12)	< 0.001
METS‐VF group						
Low (< inflection)	1.00 (Ref)	—	1.00 (Ref)	—	1.00 (Ref)	—
High (≥ inflection)	3.33 (2.52–4.49)	< 0.001	3.15 (2.32–4.38)	< 0.001	3.37 (2.36–4.96)	< 0.001
Functional limitation (continuous, per 1 unit)	1.15 (1.12–1.19)	< 0.001	1.15 (1.11–1.19)	< 0.001	1.13 (1.08–1.17)	< 0.001

### 3.4. Longitudinal Association of METS‐VF and Functional Limitations With Incident CMM

In the longitudinal cohort, both METS‐VF and functional limitations emerged as strong, independent predictors of new‐onset CMM during the follow‐up period (Table 3). After full adjustment for confounders in Model 3, participants in the high METS‐VF group (defined by the RCS inflection point) had a markedly elevated risk of developing CMM compared to those in the low METS‐VF reference group (HR = 1.77, 95% CI: 1.39–2.26, *p* < 0.001). When analyzed continuously, each 1‐unit increment in METS‐VF was significantly associated with a higher risk of incident CMM (HR = 1.18, 95% CI: 1.13–1.23, *p* < 0.001). Furthermore, continuous functional limitation also demonstrated a significant and independent association with the risk of future CMM (HR = 1.12 per 1‐unit increase, 95% CI: 1.08–1.16, *p* < 0.001).

**Table 3:** Association between METS‐VF Groups and CMM in a longitudinal follow‐up.

Variable	Model 1 HR (95% CI)	Model 1 *p*	Model 2 HR (95% CI)	Model 2 *p*	Model 3 HR (95% CI)	Model 3 *p*
METS‐VF (Continuous, per 1 unit)	1.18 (1.15–1.21)	< 0.001	1.19 (1.16–1.23)	< 0.001	1.18 (1.13–1.23)	< 0.001
METS‐VF group						
Low (< inflection)	1.00 (Ref)	—	1.00 (Ref)	—	1.00 (Ref)	—
High (≥ inflection)	2.29 (1.85–2.82)	< 0.001	2.14 (1.74–2.65)	< 0.001	1.77 (1.39–2.26)	< 0.001
Functional limitation (Continuous, per 1 unit)	1.18 (1.15–1.21)	< 0.001	1.15 (1.11–1.18)	< 0.001	1.12 (1.08–1.16)	< 0.001

### 3.5. Dose‐Response and Threshold Effect Analysis

**Figure 3:**
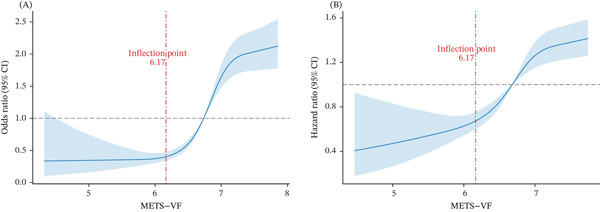
RCS analysis of METS‐VF and CMM risks. (A) Cross‐sectional association (Prevalence of CMM). (B) Longitudinal association (Incident CMM).

RCS analysis revealed a significant nonlinear dose‐response relationship between continuous METS‐VF and CMM risk (*p* for nonlinearity < 0.05; Figure 3). The inflection point was identified using the baseline prevalence data, yielding a threshold of 6.17, at which the risk of CMM began to escalate sharply. This empirically derived threshold was subsequently applied to dichotomize participants into “Low” and “High” METS‐VF risk groups for both cross‐sectional (Figure 3A) and longitudinal (Figure 3B) analyses. Even after full adjustment for all Model 3 covariates, the nonlinear associations and the predictive value of this threshold remained highly significant.

### 3.6. Association of Joint METS‐VF and Functional Limitation Grouping With CMM Incidence Risk

Table 4 presents the analysis of incident CMM risk based on the joint stratification of METS‐VF and functional limitations. In the unadjusted model (Model 1), compared to the reference group (“Neither high”), the crude HRs for the “Functional limitation only”, “High METS‐VF only”, and “Both” groups were 1.58 (95% CI: 1.05–2.37), 2.06 (95% CI: 1.57–2.70), and 3.96 (95% CI: 3.01–5.21), respectively. To rigorously control for clinical confounding, we focused on the fully adjusted model (Model 3). Notably, after adjusting for demographics, lifestyle, baseline comorbidities, renal function, and medication use, the independent association for the “Functional limitation only” group was attenuated and became statistically nonsignificant (HR = 1.24, 95% CI: 0.77–1.99, P = 0.378). However, the “High METS‐VF only” group maintained a robust association with incident CMM (HR = 1.62, 95% CI: 1.20–2.19, *p* = 0.002). Most importantly, the “Both” group retained the highest overall risk of developing CMM (HR = 2.43, 95% CI: 1.78–3.31, *p* < 0.001).

**Table 4:** Associations among baseline METS‐VF and functional limitations with CMM.

Joint risk category	Model 1 HR (95% CI)	Model 1 *p*	Model 2 HR (95% CI)	Model 2 *p*	Model 3 HR (95% CI)	Model 3 *p*
Neither high (Reference)	1.00 (Ref)	—	1.00 (Ref)	—	1.00 (Ref)	—
Functional limitation only	1.58 (1.05–2.37)	0.028	1.48 (0.98–2.22)	0.061	1.24 (0.77–1.99)	0.378
High METS‐VF only	2.06 (1.57–2.70)	< 0.001	1.99 (1.52–2.62)	< 0.001	1.62 (1.20–2.19)	0.002
Both high	3.96 (3.01–5.21)	< 0.001	3.46 (2.61–4.57)	< 0.001	2.43 (1.78–3.31)	< 0.001

### 3.7. Subgroup Analysis

**Figure 4:**
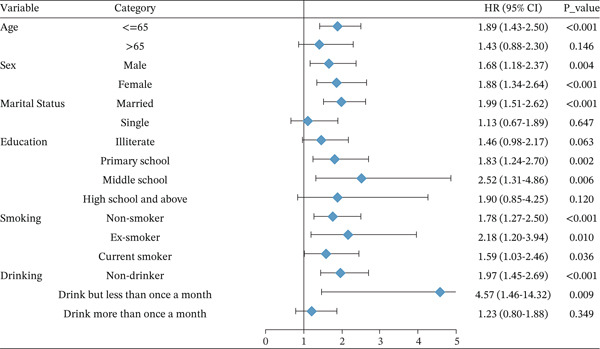
Subgroup analysis of the combined effect of METS‐VF and functional limitation on CMM risk.

Subgroup analyses were conducted to evaluate the robustness of the association between high METS‐VF and incident CMM (Figure 4). The positive association remained significant across both sexes (Men: HR = 1.68, 95% CI: 1.18–2.37; Women: HR = 1.88, 95% CI: 1.34–2.64). Notably, the association was significant in participants aged ≤ 65 years (HR = 1.89, 95% CI: 1.43–2.50, *p* < 0.001) but did not reach statistical significance in those > 65 years (HR = 1.43, 95% CI: 0.88–2.30, *p* = 0.146). Similarly, high METS‐VF significantly predicted incident CMM in nondrinkers (HR = 1.97, *p* < 0.001) but not in frequent drinkers (HR = 1.23, *p* = 0.349). Additionally, significant predictive values were prominently observed among exsmokers (HR = 2.18, 95% CI: 1.20–3.94), married individuals (HR = 1.99, 95% CI: 1.51–2.62), and those with middle school education (HR = 2.52, 95% CI: 1.31–4.86).

### 3.8. Sensitivity Analyses

The results of the three predefined sensitivity analyses (Table S1) confirmed the exceptional robustness of our primary findings. In the treatment‐naive cohort (Panel A), the simultaneous presence of high METS‐VF and functional limitations continued to exhibit the highest risk for incident CMM (HR = 2.49, 95% CI: 1.72–3.61, *p* < 0.001). Similarly, in the lag analysis (Panel B), the “Both high” group maintained a profound joint effect (HR = 2.33, 95% CI: 1.67–3.25, *p* < 0.001). Finally, within the “super‐clean” subcohort (Panel C), the joint presence of these two factors remained a powerful predictor of incident CMM (HR = 2.53, 95% CI: 1.47–4.34, *p* < 0.001). Furthermore, continuous METS‐VF and functional limitation scores consistently maintained their independent predictive values across all three sensitivity models (all *p* < 0.001).

### 3.9. Discriminative Power Analysis

**Figure 5:**
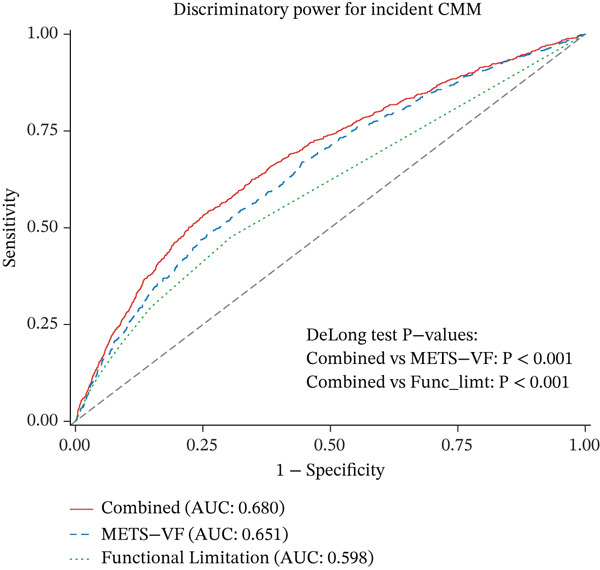
Comparison of ROC curves for predicting CMM risk between METS‐VF and functional limitation.

To evaluate the predictive performance for incident CMM, we constructed ROC curves and calculated the AUC (Figure 5). The model based solely on METS‐VF yielded an AUC of 0.651, demonstrating greater discriminatory power than the model based on functional limitations alone (AUC = 0.598). Notably, the combined model integrating both risk factors achieved the highest predictive accuracy (AUC = 0.680). DeLong′s test confirmed that this combined model showed a statistically significant improvement over both the METS‐VF‐only model (*p* < 0.001) and the functional limitation‐only model (*p* < 0.001). Furthermore, to quantify the incremental predictive value beyond traditional ROC evaluation, we calculated the continuous net reclassification improvement (NRI) and integrated discrimination improvement (IDI) (Table 5). The addition of both METS‐VF and functional limitation to the fully adjusted baseline clinical model resulted in a significant improvement in risk reclassification (Continuous NRI = 0.081, 95% CI: 0.030–0.159, *p* < 0.001). Additionally, the overall discrimination of the model was significantly enhanced, with an IDI of 0.012 (95% CI: 0.002–0.024, *p* = 0.027).

**Table 5:** Incremental predictive value of METS‐VF and functional limitation for the risk of incident CMM.

Metrics	Estimate	95% CI	*p*
Continuous NRI	0.081	0.030–0.159	< 0.001
IDI	0.012	0.002–0.024	0.027

Abbreviations: IDI, Integrated discrimination improvement; NRI, Net reclassification improvement.

### 3.10. Mediation Effect of Systemic Inflammation

**Figure 6:**
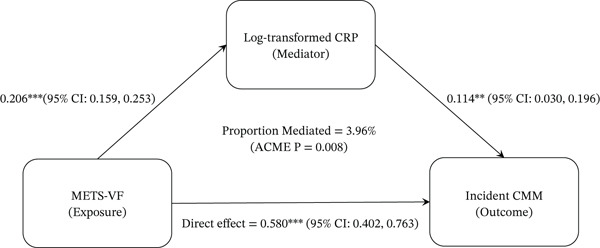
Mediation path diagram evaluating the role of systemic inflammation in the association between METS‐VF and incident CMM.

Based on the observed baseline differences in inflammatory markers, we conducted a mediation analysis to investigate whether CRP mediates the association between METS‐VF and the incidence of CMM. The analysis revealed a statistically significant indirect effect of METS‐VF on CMM risk through elevated CRP levels (Average causal mediation effect [ACME] *p* = 0.008). Specifically, approximately 3.96% of the total association between METS‐VF and incident CMM was mediated by CRP (Figure 6). These empirical data corroborate that systemic inflammation serves as a significant biological bridge linking visceral adiposity to the development of CMM.

## 4. Discussion

This study is the first to explore the joint effect of METS‐VF and functional limitations on the risk of CMM incidence. Our fully adjusted models robustly demonstrate that compared to the low‐risk reference group, high METS‐VF alone was independently associated with an elevated risk of incident CMM (HR = 1.62, 95% CI: 1.20–2.19). Most notably, the simultaneous presence of both high METS‐VF and functional limitations resulted in the highest risk (HR = 2.43, 95% CI: 1.78–3.31), demonstrating a profound joint effect.

Our findings align with previous literature. For instance, Feng et al. [10] demonstrated that METS‐VF is a reliable and effective predictor of Type 2 diabetes mellitus (T2DM) incidence in the Chinese population. Analysis from the NHANES study indicated that METS‐VF is a reliable, noninvasive, and cost‐effective predictor of visceral obesity, linearly and positively correlated with atherosclerotic cardiovascular disease, serving as an accurate indicator of disease risk [26]. Zhang et al. [27] studied 5127 participants in Southwest China to explore the relationship between METS‐VF and hypertension across different ethnicities, finding a significant positive correlation between hypertension risk and METS‐VF levels; as METS‐VF increased, the onset of hypertension occurred earlier. Existing literature highlights the association between CMDs and functional limitation. Diabetes has been reported to increase the risk of disability by 65%–82% [28], and coronary heart disease by 57% [29]. Furthermore, stroke has been identified as having the most substantial impact on functional impairment [30]. Consistent with Ni et al. [31], our longitudinal data demonstrated that functional limitations themselves (as a baseline exposure) also predicted future CMM occurrence. Although our study provides robust evidence within the Chinese aging population, the generalizability of these findings should be interpreted in light of inherent heterogeneities across different cohorts. First, regarding population characteristics, ethnic differences in adipose tissue distribution are substantial. Asian populations inherently tend to accumulate more visceral fat at lower BMI levels compared to western populations [32, 33]. Consequently, the specific threshold of METS‐VF associated with elevated CMM risk (e.g., our empirically derived inflection point of 6.17) may differ significantly from those in European or North American cohorts, limiting the direct extrapolation of specific cutoffs across diverse ethnicities [34]. Second, measurement discrepancies across studies can contribute to variations in reported effect sizes. Although METS‐VF serves as a validated cost‐effective surrogate for visceral adiposity, studies utilizing direct anatomical imaging techniques (such as MRI or DEXA) might report different magnitudes of association due to their precise quantification of ectopic fat [35]. Similarly, our assessment of functional limitations relied on self‐reported ADL and IADL scales. Studies employing objective physical performance metrics (e.g., gait speed, grip strength, or the Short Physical Performance Battery) might capture different dimensions of physical frailty, potentially yielding discrepant predictive values [36, 37].

METS‐VF and functional limitations may be associated with CMM through multiple biological pathways. First is the superposition of inflammatory burden: high METS‐VF is a key source of systemic chronic low‐grade inflammation. VAT is highly metabolically active, secreting pro‐inflammatory cytokines (such as IL‐6 and TNF‐*α*), ultimately contributing to chronic low‐grade inflammation [38]. Concurrently, functional limitation—and the resulting lack of physical activity—acts as an independent pro‐inflammatory driver [39]. When both coexist, their inflammatory burdens superimpose, jointly accelerating vascular endothelial injury, atherosclerosis, and islet cell dysfunction—all core pathologies of CMM. Specifically, at the molecular level, this inflammatory cascade operates through precise pathogenic pathways. Elevated TNF‐*α* directly impairs insulin signaling by promoting the serine phosphorylation of insulin receptor substrate‐1 (IRS‐1), thereby inducing severe peripheral metabolic resistance [40]. Simultaneously, IL‐6 drives the hepatic synthesis of CRP. Crucially, recent evidence emphasizes that CRP functions not merely as a passive biomarker, but as an active atherogenic mediator. It directly interacts with the endothelium to downregulate endothelial nitric oxide synthase (eNOS) expression and bioactivity, fundamentally depleting nitric oxide (NO) availability. This process, coupled with the upregulation of vascular adhesion molecules (e.g., VCAM‐1), actively accelerates endothelial apoptosis and atherosclerotic plaque progression [41, 42]. Furthermore, prolonged exposure to this inflammatory cytokine milieu directly induces pancreatic *β*‐cell apoptosis, irreversibly damaging glucose homeostasis [43]. Importantly, our formal mediation analysis quantified that systemic inflammation plays a mediating role in this process, with elevated CRP levels accounting for approximately 3.96% of the total effect of METS‐VF on incident CMM (*p* = 0.008). This aligns with very recent evidence demonstrating that the adverse cardiometabolic effects of METS‐VF are significantly mediated through systemic inflammatory markers like CRP [44]. This objective data firmly substantiates our hypothesis that visceral adiposity acts not merely as a passive energy reservoir, but as an active endocrine organ fostering a systemic low‐grade inflammatory state [45], which subsequently accelerates the progression toward CMM.

Second is the exacerbation of insulin resistance: High METS‐VF mainly reflects hepatic and central insulin resistance, whereas functional limitations are linked to decreased peripheral glucose uptake capacity, exacerbating peripheral insulin resistance [46, 47]. The simultaneous deterioration of central and peripheral insulin resistance is catastrophic for glucose homeostasis, dramatically elevating the risk of diabetes and related metabolic disorders. To further elucidate how functional limitations act as an upstream contributor of this peripheral metabolic collapse, two specific muscle‐centric pathways must be highlighted. The first key mechanism is the disruption of muscle‐organ cross‐talk and myokine signaling. Skeletal muscle is now recognized as an active endocrine organ; functional limitations and the resulting sedentary behavior contribute to “myokine deficiency” [48]. The reduced secretion of muscle‐derived factors, such as irisin and IL‐15, impairs the “browning” of white adipose tissue and diminishes systemic glucose clearance capacity, directly fostering a prodiabetic environment. Furthermore, this is compounded by sarcopenic pathways. Chronic functional impairment triggers muscle atrophy and sarcopenia, which substantially reduces the primary site for insulin‐stimulated glucose uptake. This loss of metabolic sink creates a state of “metabolic inflexibility,” which is linked to compensatory hyperinsulinemia and subsequent vascular endothelial damage [49].

Moreover, psycho‐neuro‐endocrine mechanisms are implicated in this association. Functional limitations in older adults can contribute to health anxiety, fear of dependency, and social isolation. Consequently, these factors increase vulnerability to psychological distress, including depression and anxiety [50, 51]. The loss of autonomy and social isolation inherent in functional limitation act as chronic stressors that overactivate the hypothalamic‐pituitary‐adrenal (HPA) axis. Persistent elevation of cortisol levels not only promotes visceral fat redistribution (further elevating METS‐VF) but also directly induces hypertension and dyslipidemia, thereby accelerating the cluster of cardiometabolic conditions that define CMM [52]. This endocrine disruption is further compounded by visceral adiposity itself. Individuals with high METS‐VF have adipocytes and infiltrating macrophages distributed in fat that are capable of secreting inflammatory mediators [53, 54]. The resulting inflammatory cytokines are associated with changes in neural plasticity and brain circuits, disrupt neurotransmitter metabolism and function, and stimulate the neuroendocrine system, contributing to more severe psychological stress [55]. Moreover, a mutual promotion relationship exists between functional limitations and high METS‐VF: reduced physical activity contributes to lowered energy expenditure, promoting a positive energy balance, which in turn exacerbates visceral fat accumulation and dysfunction [56], manifested as an elevated METS‐VF score. Conversely, visceral fat accumulation releases free fatty acids, pro‐inflammatory cytokines, and reactive oxygen species, significantly affecting muscle mass and strength loss, promoting muscle atrophy, and further aggravating functional limitations [57, 58]. Collectively, these pathways establish a vicious bidirectional cycle where CMM is a risk factor for functional decline, and functional limitations, in turn, provide the physiological and psychological substrate for the rapid progression of multimorbidity.

Beyond the biological plausibility of these pathways, the statistical robustness of our findings is further underscored by a series of rigorous sensitivity analyses. By performing a lag analysis that excluded cases occurring within the first 2 years of follow‐up, we minimized the potential for reverse causation, thereby strengthening the temporal sequence between baseline exposures and incident CMM. Furthermore, the persistent significance observed after excluding all baseline medication users effectively ruled out pharmacological confounding as a driver of our results. Perhaps most convincingly, our findings remained stable even in a “super‐clean” cohort of individuals entirely free of any single cardiometabolic component at baseline. This suggests that the joint impact of elevated visceral adiposity and functional impairment is a fundamental correlate of multi‐systemic collapse, rather than merely a reflection of preexisting disease progression. These multifaceted validations collectively enhance the internal validity of our study and provide high‐level evidence for the clinical integration of METS‐VF and functional screening.

In subgroup analyses, several profound pathophysiological and demographic heterogeneities were observed, providing deeper mechanistic insights into the risk profiles. First, regarding age and sex heterogeneities, the association was highly significant in middle‐aged populations (≤ 65 years, HR = 1.89) but lost statistical significance in the advanced elderly (> 65 years). This discrepancy is pathophysiologically sound. In middle‐aged individuals, visceral adiposity acts as a primary aggressive contributor to metabolic dysfunction and systemic inflammation. Conversely, in the advanced elderly population, the aging process itself is accompanied by profound systemic immunosenescence, widespread organ decline, and a multitude of competing morbidity risks. These overwhelming age‐related baseline burdens may dilute or mask the specific predictive value of METS‐VF and physical limitations [59]. Furthermore, women exhibited a slightly stronger effect (HR = 1.88) compared to men (HR = 1.68). This is attributable to postmenopausal metabolic shifts; the sharp decline in estrogen drastically redistributes adipose tissue to the visceral cavity, making postmenopausal women acutely vulnerable to the lipotoxic impacts of elevated METS‐VF [60]. Second, lifestyle factors (smoking and drinking) revealed striking metabolic interactions. Notably, the highest risk among smoking categories was observed in exsmokers (HR = 2.18), surpassing both nonsmokers and current smokers. This robustly aligns with the well‐documented “post‐cessation weight gain” (PCWG) phenomenon, where smoking cessation is frequently accompanied by a rapid, transient surge in visceral fat accumulation and worsened peripheral insulin resistance, thereby exacerbating the METS‐VF‐driven metabolic collapse [61]. Regarding alcohol consumption, the predictive value of METS‐VF was highly significant in nondrinkers (HR = 1.97, *p* < 0.001) but was lost in frequent drinkers (> once a month, *p* = 0.349). This statistical divergence is highly consistent with recent pathophysiological insights. In nondrinkers, visceral adiposity operates as the unconfounded primary driver of cardiometabolic deterioration. Conversely, chronic and frequent alcohol consumption exerts an independent, profound hepatotoxic and metabolic impact—specifically by promoting alcohol‐associated steatotic liver disease, severe oxidative stress, and direct hepatic insulin resistance [62]. This aggressive, alcohol‐induced pathogenesis acts as a powerful competing risk pathway that essentially overrides and overshadows the specific lipotoxic mechanisms evaluated by the METS‐VF score alone [63]. Third, socioeconomic and familial environments significantly modified the risk. The effect was pronounced in married individuals (HR = 1.99) but nonsignificant in single participants. This likely reflects “spousal concordance” in cardiometabolic risks, where married couples share obesogenic environments, sedentary behaviors, and dietary patterns that jointly amplify the detriments of high METS‐VF [64]. Conversely, single older adults (often widowed) generally suffer from higher baseline frailty, which acts as a competing risk [65]. Lastly, the risk was significant in individuals with middle school education (HR = 2.52) but not in those with high school education or above, highlighting that lower educational attainment—and its correlation with suboptimal health literacy and delayed preventive care—amplifies the consequences of metabolic dysregulation [66].

Translating these pathophysiological findings into clinical practice, our study highlights the substantial public health value of this joint evaluation. As evidenced by the significant improvements in risk reclassification (Continuous NRI = 0.081; IDI = 0.012), integrating METS‐VF and functional limitation provides a superior clinical tool for identifying “hidden” high‐risk individuals who might otherwise be missed by traditional risk models. Given this strong predictive value, integrating METS‐VF into routine clinical workflows presents a highly feasible and cost‐effective strategy, particularly in primary care and resource‐limited settings. Crucially, METS‐VF calculation utilizes only routinely available and inexpensive clinical parameters (BMI, FBG, triglycerides, and HDL‐C), thereby completely bypassing the need for costly and largely inaccessible imaging modalities like MRI or CT. Compared to established cardiovascular risk assessment tools (such as the Framingham risk score or ASCVD risk estimator) that primarily rely on chronological age and standard lipid profiles [67], METS‐VF offers a distinct complementary advantage by specifically capturing the profound lipotoxic burden and early metabolic dysfunction associated with visceral adiposity prior to the onset of overt clinical disease. In terms of practical implementation, METS‐VF can serve as an efficient initial screening metric. Regarding monitoring frequency, we propose that METS‐VF be evaluated annually for the general aging population during routine health check‐ups. However, for individuals exhibiting borderline metabolic disturbances (e.g., prediabetes) or those approaching the high‐risk threshold, semi‐annual monitoring is recommended to capture rapid metabolic shifts. When an individual is identified as exceeding the high‐risk threshold (e.g., METS − VF ≥ 6.17), primary care physicians should immediately implement a multidimensional screening protocol, seamlessly triggering a rapid, standardized functional capacity assessment. This stepwise, joint evaluation strategy allows for the precise triaging of aging populations, guiding early, targeted multidomain interventions—such as structured exercise prescriptions, intensive metabolic control, and sarcopenia prevention—ultimately delaying or preventing the complex onset of CMM [68].

## 5. Strengths and Limitations

This study possesses several significant strengths. First, it is based on CHARLS, a large nationally representative prospective longitudinal cohort study, enabling both cross‐sectional and longitudinal analyses to reliably assess the temporal association of METS‐VF and functional limitations with CMM risk. Second, we employed the novel biomarker METS‐VF, which outperforms traditional obesity indices in assessing visceral fat function. Third, the definition of CMM adopted a comprehensive approach using multisource information, and ADL and IADL were comprehensively assessed to define functional limitation.

However, several key methodological limitations must be explicitly acknowledged. First, regarding the timing of exposure assessment, our study relied exclusively on a single baseline measurement. Consequently, we were unable to capture the dynamic trajectories or cumulative duration of elevated METS‐VF and functional decline over the follow‐up period, which may introduce regression dilution bias. Second, although we performed rigorous sensitivity analyses (e.g., lag analysis and exclusion of baseline medication users) to validate our findings, our treatment of missing data relied on a complete‐case approach. The exclusion of individuals with missing baseline or follow‐up data, rather than utilizing multiple imputation, may introduce a degree of selection bias. Third, although our joint stratification robustly demonstrated the highest risk in the “Both high” group, formal testing did not yield a statistically significant multiplicative interaction. Thus, the observed association is strictly interpreted as a powerful cumulative joint effect rather than a true biological synergism. Fourth, some CMM diagnoses and functional limitation assessments relied on participant self‐reports, potentially introducing recall bias. Fifth, our study population consists exclusively of Chinese adults aged 45 years and older. Consequently, inherent differences in demographic structure, genetic background, and lifestyle factors may significantly limit the generalizability of our findings to younger populations or other ethnic groups. Therefore, these results must be extrapolated with caution. Due to the inherent observational nature of the CHARLS cohort design, our study can only establish temporal associations rather than definitive causal relationships between the exposures and incident CMM. Future well‐designed mechanistic studies and randomized controlled trials across diverse cohorts are required to validate these findings. Lastly, it is important to note that the wide 95% CIs observed in certain subgroup analyses (e.g., specific alcohol consumption categories) indicate limited statistical precision. This is likely driven by the relatively small sample sizes and low event rates within these specific strata. Consequently, although the positive associations remain statistically significant, the exact magnitude of these specific effect sizes should be interpreted with caution.

## 6. Conclusion

Our findings indicate that the combined effect of high METS‐VF and functional limitations is significantly associated with an increased risk of developing CMM. The study confirms that METS‐VF (representing visceral fat dysfunction) and functional limitations are not only independent predictors of CMM but also exert a powerful joint effect (HR = 2.43) when present simultaneously. Therefore, early detection and intervention regarding high METS‐VF status and physical functional limitations are of great significance for the prevention and treatment of CMM. In clinical risk assessment, considering both METS‐VF and functional limitation status can significantly improve CMM prediction efficacy (AUC increased from 0.651 with a single indicator to 0.680 with the joint model) and help more accurately identify the highest‐risk population (i.e., the “Both high” group) at an early stage.

## Author Contributions

Xuhui Chen and Jianhui Liu contributed to the conception or design of the work. Xuhui Chen, Ying Wang, Yulian He, Huihui Chen, Honghua Ye, and Jiaofen Wu contributed to the acquisition, analysis, or interpretation of data for the work. Xuhui Chen wrote the first draft of the manuscript and Jianhui Liu revised the manuscript.

## Funding

This work was supported by the Medical and Health Research Project of Zhejiang Province, WKJ‐ZJ‐2446 and the Ningbo Key Research and Development Program, 2024Z232.

## Disclosure

This manuscript has not been published or presented elsewhere in part or in entirety, and is not under consideration by another journal. All the authors have approved the manuscript and agree with submission to your esteemed journal.

## Ethics Statement

The studies involving humans were approved by Lihuili Hospital Ethics Review Board (KY2025ML099). The studies were conducted in accordance with the local legislation and institutional requirements. Written informed consent for participation was not required from the participants or the participants′ legal guardians/next of kin in accordance with the national legislation and institutional requirements.

## Conflicts of Interest

The authors declare no conflicts of interest.

## Supporting information


**Supporting Information** Additional supporting information can be found online in the Supporting Information section. Table S1 Sensitivity analyses evaluating the robustness of the associations between METS‐VF, functional limitation, and incident CMM.

## Data Availability

The datasets analyzed in this study are publicly accessible from the China Health and Retirement Longitudinal Study (CHARLS) repository at http://charls.pku.edu.cn. Additional data requests may be directed to the corresponding author.

## References

[1] Jenkins D. J. A. , Willett W. C. , Yusuf S. , Hu F. B. , Glenn A. J. , Liu S. , Mente A. , Miller V. , Bangdiwala S. I. , Gerstein H. C. , Sieri S. , Ferrari P. , Patel A. V. , McCullough M. L. , le Marchand L. , Freedman N. D. , Loftfield E. , Sinha R. , Shu X. O. , Touvier M. , Sawada N. , Tsugane S. , van den Brandt P. A. , Shuval K. , Khan T. A. , Paquette M. , Sahye-Pudaruth S. , Patel D. , Siu T. F. Y. , Srichaikul K. , Kendall C. W. C. , Sievenpiper J. L. , Balachandran B. , Zurbau A. , Wang X. , Liang F. , and Yang W. , Association of Glycaemic Index and Glycaemic Load With Type 2 Diabetes, Cardiovascular Disease, Cancer, and All-Cause Mortality: A Meta-Analysis of Mega Cohorts of More Than 100?000 participants, Lancet Diabetes & Endocrinology. (2024) 12, no. 2, 107–118, 10.1016/S2213-8587(23)00344-3, 38272606. 38272606

[2] Xiao D. , Sun H. , Chen L. , Li X. , Huo H. , Zhou G. , Zhang M. , and He B. , Assessment of Six Surrogate Insulin Resistance Indexes for Predicting Cardiometabolic Multimorbidity Incidence in Chinese middle‐aged and Older Populations: Insights From the China Health and Retirement Longitudinal study, Diabetes/Metabolism Research and Reviews. (2024) 40, no. 1, e3764, 10.1002/dmrr.3764, 38287717. 38287717

[3] Fan J. , Sun Z. , Yu C. , Guo Y. , Pei P. , Yang L. , Chen Y. , du H. , Sun D. , Pang Y. , Zhang J. , Gilbert S. , Avery D. , Chen J. , Chen Z. , Lyu J. , Li L. , and China Kadoorie Biobank Collaborative Group , Multimorbidity Patterns and Association With Mortality in 0.5 Million Chinese adults, Chinese Medical Journal. (2022) 135, no. 6, 648–657, 10.1097/CM9.0000000000001985, 35191418. 35191418 PMC9276333

[4] Sakakibara B. M. , Obembe A. O. , and Eng J. J. , The Prevalence of Cardiometabolic Multimorbidity and its Association With Physical Activity, Diet, and Stress in Canada: Evidence From a Population-Based Cross-Sectional study, BMC Public Health. (2019) 19, no. 1, 10.1186/s12889-019-7682-4, 31651286. PMC6814029 31651286

[5] Kivimäki M. , Kuosma E. , Ferrie J. E. , Luukkonen R. , Nyberg S. T. , Alfredsson L. , Batty G. D. , Brunner E. J. , Fransson E. , Goldberg M. , Knutsson A. , Koskenvuo M. , Nordin M. , Oksanen T. , Pentti J. , Rugulies R. , Shipley M. J. , Singh-Manoux A. , Steptoe A. , Suominen S. B. , Theorell T. , Vahtera J. , Virtanen M. , Westerholm P. , Westerlund H. , Zins M. , Hamer M. , Bell J. A. , Tabak A. G. , and Jokela M. , Overweight, Obesity, and Risk of Cardiometabolic Multimorbidity: Pooled Analysis of Individual-Level Data for 120?813 Adults From 16 Cohort Studies From the USA and Europe, Lancet Public Health. (2017) 2, no. 6, e277–e285, 10.1016/S2468-2667(17)30074-9, 28626830. 28626830 PMC5463032

[6] Ibrahim M. M. , Subcutaneous and Visceral Adipose Tissue: Structural and Functional differences, Obesity Reviews: An Official Journal of the International Association for the Study of Obesity. (2010) 11, no. 1, 11–18, 10.1111/j.1467-789X.2009.00623.x. 19656312

[7] Nevill A. M. , Stewart A. D. , Olds T. , and Holder R. , Relationship Between Adiposity and Body Size Reveals Limitations of BMI, American Journal of Physical Anthropology. (2006) 129, no. 1, 151–156, 10.1002/ajpa.20262. 16270304

[8] Bello-Chavolla O. Y. , Antonio-Villa N. E. , Vargas-Vázquez A. , Viveros-Ruiz T. L. , Almeda-Valdes P. , Gomez-Velasco D. , Mehta R. , Elias-López D. , Cruz-Bautista I. , Roldán-Valadez E. , Martagón A. J. , and Aguilar-Salinas C. A. , Metabolic Score for Visceral Fat (METS-VF), A Novel Estimator of Intra-Abdominal Fat Content and Cardio-Metabolic Health, Clinical Nutrition. (2020) 39, no. 5, 1613–1621, 10.1016/j.clnu.2019.07.012, 31400997. 31400997

[9] Feng Y. , Yang X. , Li Y. , Wu Y. , Han M. , Qie R. , Huang S. , Wu X. , Zhang Y. , Liu D. , Hu F. , Zhang M. , Yang Y. , Shi X. , Lu J. , Zhao Y. , and Hu D. , Metabolic Score for Visceral Fat: A Reliable Indicator of Visceral Obesity for Predicting Risk for Hypertension, Nutrition. (2022) 93, 111443, 10.1016/j.nut.2021.111443. 34563934

[10] Feng Y. , Yang X. , Li Y. , Wu Y. , Han M. , Qie R. , Huang S. , Wu X. , Zhang Y. , Zhang J. , Hu H. , Yuan L. , Li T. , Liu D. , Hu F. , Zhang M. , Zeng Y. , Luo X. , Lu J. , Sun L. , Hu D. , and Zhao Y. , Metabolic Score for Visceral Fat: A Novel Predictor for the Risk of Type 2 Diabetes Mellitus, British Journal of Nutrition. (2022) 128, no. 6, 1029–1036, 10.1017/S0007114521004116, 34632975. 34632975

[11] Wu Y. , Xu J. , Gao Y. , and Zheng J. , The Relationship Between Health Behaviors and Quality of Life: The Mediating Roles of Activities of Daily Living and Psychological distress, Frontiers in Public Health. (2024) 12, 1398361, 10.3389/fpubh.2024.1398361, 38864012. 38864012 PMC11165072

[12] Bessen S. , Garcia Morales E. E. , Zhang W. , Martinez-Amezcua P. , Umoh M. , Cudjoe T. K. M. , Schrack J. A. , and Reed N. S. , Hearing Loss, Difficulty With Activities of Daily Living, and Experience of Consequences of related Unmet Needs in Older Adults: A Cross-Sectional Analysis, American Journal of Audiology. (2025) 34, no. 1, 127–138, 10.1044/2024_AJA-24-00183, 39932395. 39932395

[13] Devassy S. M. and Scaria L. , Prevalence and Risk Factors for Falls in Community-Dwelling Older Population in Kerala; Results from a Cross Sectional Survey, Heliyon. (2023) 9, no. 8, e18737, 10.1016/j.heliyon.2023.e18737, 37593613. 37593613 PMC10428049

[14] Liu H. , Zhang X. , Chen B. , Fang B. , Lou V. W. Q. , and Hu J. , The Differential Impact of Multimorbidity Patterns and Subsequent Accumulation on Longitudinal Trajectories of Physical Function Decline in a Population-Based Cohort of Older People, Journals of Gerontology. Series A, Biological Sciences and Medical Sciences. (2022) 77, no. 8, 1629–1636, 10.1093/gerona/glab384. 34951651

[15] Hu Z. , Zheng B. , Kaminga A. C. , Zhou F. , and Xu H. , Association Between Functional Limitations and Incident Cardiovascular Diseases and All-Cause Mortality Among the Middle-Aged and Older Adults in China: A Population-Based Prospective Cohort Study, Frontiers in Public Health. (2022) 10, 751985, 10.3389/fpubh.2022.751985, 35223720. 35223720 PMC8873112

[16] Qiao Y. , Liu S. , Li G. , Lu Y. , Wu Y. , Ding Y. , and Ke C. , Role of Depressive Symptoms in Cardiometabolic Diseases and Subsequent Transitions to All-Cause Mortality: An Application of Multistate Models in a Prospective Cohort study, Stroke and Vascular Neurology. (2021) 6, no. 4, 511–518, 10.1136/svn-2020-000693, 33741743. 33741743 PMC8717791

[17] Martin Ginis K. A. , van der Ploeg H. P. , Foster C. , Lai B. , McBride C. B. , Ng K. , Pratt M. , Shirazipour C. H. , Smith B. , Vásquez P. M. , and Heath G. W. , Participation of People Living With Disabilities in Physical Activity: A Global perspective, Lancet. (2021) 398, no. 10298, 443–455, 10.1016/S0140-6736(21)01164-8, 34302764. 34302764

[18] He L. , Lin C. , Tu Y. , Yang Y. , Lin M. , Tu H. , and Li J. , Correlation of Cardiometabolic Index and Sarcopenia With Cardiometabolic Multimorbidity in Middle-Aged and Older Adult: A Prospective Study, Frontiers in Endocrinology. (2024) 15, 1387374, 10.3389/fendo.2024.1387374, 38863933. 38863933 PMC11165091

[19] Li H. , Zheng D. , Li Z. , Wu Z. , Feng W. , Cao X. , Wang J. , Gao Q. , Li X. , Wang W. , Hall B. J. , Xiang Y. T. , and Guo X. , Association of Depressive Symptoms With Incident Cardiovascular Diseases in Middle-Aged and Older Chinese Adults, JAMA Network Open. (2019) 2, no. 12, e1916591, 10.1001/jamanetworkopen.2019.16591, 31800066. 31800066 PMC6902756

[20] 2. Classification and Diagnosis of Diabetes:Standards of Medical Care in Diabetes-2022, Diabetes Care. (2022) 45, no. Supplement_1, S17–S38, 10.2337/dc22-S002, 34964875. 34964875

[21] Chobanian A. V. , Bakris G. L. , Black H. R. , Cushman W. C. , Green L. A. , Izzo JL Jr , Jones D. W. , Materson B. J. , Oparil S. , Wright JT Jr , Roccella E. J. , National Heart, Lung, and Blood Institute Joint National Committee on Prevention, Detection, Evaluation, and Treatment of High Blood Pressure , and National High Blood Pressure Education Program Coordinating Committee , The Seventh Report of the Joint National Committee on Prevention, Detection, Evaluation, and Treatment of High Blood Pressure: The JNC 7 Report, Jama. (2003) 289, no. 19, 2560–2572, 10.1001/jama.289.19.2560, 12748199. 12748199

[22] Bello-Chavolla O. Y. , Almeda-Valdes P. , Gomez-Velasco D. , Viveros-Ruiz T. , Cruz-Bautista I. , Romo-Romo A. , Sánchez-Lázaro D. , Meza-Oviedo D. , Vargas-Vázquez A. , Campos O. A. , Sevilla-González M. D. R. , Martagón A. J. , Hernández L. M. , Mehta R. , Caballeros-Barragán C. R. , and Aguilar-Salinas C. A. , METS-IR, A Novel Score to Evaluate Insulin Sensitivity, is Predictive of Visceral Adiposity and Incident Type 2 diabetes, European Journal of Endocrinology. (2018) 178, no. 5, 533–544, 10.1530/EJE-17-0883, 29535168. 29535168

[23] Katz S. , Ford A. B. , Moskowitz R. W. , Jackson B. A. , and Jaffe M. W. , Studies of illness in the Aged, Jama. (1963) 185, no. 12, 914–919, 10.1001/jama.1963.03060120024016. 14044222

[24] Lawton M. P. and Brody E. M. , Assessment of Older People: Self-Maintaining and Instrumental Activities of Daily Living, Gerontologist. (1969) 9, 3 Part 1, 179–186, 10.1093/geront/9.3_Part_1.179, 5349366. 5349366

[25] Zhao W. , Si Y. , Li X. , Zhao Y. , Jia S. , and Dong B. , Association of Allostatic Load With Functional Disability in the China Health and Retirement Longitudinal Study, Journal of Nutrition, Health & Aging. (2024) 28, no. 11, 100367, 10.1016/j.jnha.2024.100367, 39341031. PMC12879250 39341031

[26] Wu S. , Jiang S. , Yao Y. , and Li M. , Association Between Visceral Fat Metabolism Score and Atherosclerotic Cardiovascular Disease: Evidence From NHANES 2005 to 2016, Coronary Artery Disease. (2025) 36, no. 8, 654–661, 10.1097/MCA.0000000000001545, 40521653. 40521653

[27] Zhang F. , Wang Y. , Zhou J. , Yu L. , Wang Z. , Liu T. , and Yu Y. , Association between Metabolic Score for Visceral Fat and The Risk of Hypertension in Different Ethnic Groups: A Prospective Cohort Study in Southwest China, Frontiers in Endocrinology. (2024) 15, 1302387, 10.3389/fendo.2024.1302387, 38562413. 38562413 PMC10982387

[28] Wong E. , Backholer K. , Gearon E. , Harding J. , Freak-Poli R. , Stevenson C. , and Peeters A. , Diabetes and Risk of Physical Disability in Adults: A Systematic Review and meta-analysis, Lancet Diabetes & Endocrinology. (2013) 1, no. 2, 106–114, 10.1016/S2213-8587(13)70046-9, 24622316. 24622316

[29] Kodera R. , Fujihara K. , Koyama T. , Shiozaki H. , Mutsuma Y. , Yagyuda N. , Hatta M. , Tsuruoka K. , Takeda Y. , Araki A. , and Sone H. , Impact of a History of Cardiovascular Disease and Physical Activity Habits on the Incidence of Functional disability, Scientific Reports. (2023) 13, no. 1, 10.1038/s41598-023-47913-z, 38012261. PMC10682401 38012261

[30] Hu W. H. , Liu Y. Y. , Yang C. H. , Zhou T. , Yang C. , Lai Y. S. , Liao J. , and Hao Y. T. , Developing and Validating a Chinese Multimorbidity-Weighted Index for Middle-Aged and Older Community-Dwelling individuals, Age and Ageing. (2022) 51, no. 2, afab274, 10.1093/ageing/afab274, 35211718. 35211718

[31] Ni P. , Wang F. , Liu L. , Ge M. , and Hu X. , Association of Functional Disability With Cardiometabolic Disease Status in a National Cohort study, Experimental Gerontology. (2025) 206, 112771, 10.1016/j.exger.2025.112771, 40318705. 40318705

[32] Lim U. , Ernst T. , Buchthal S. D. , Latch M. , Albright C. L. , Wilkens L. R. , Kolonel L. N. , Murphy S. P. , Chang L. , Novotny R. , and le Marchand L. , Asian women Have Greater Abdominal and Visceral Adiposity Than Caucasian Women With Similar Body Mass index, Nutrition & Diabetes. (2011) 1, no. 5, 10.1038/nutd.2011.2, 23449381. PMC3302135 23449381

[33] Wulan S. N. , Westerterp K. R. , and Plasqui G. , Ethnic Differences in Body Composition and the Associated Metabolic Profile: A Comparative Study Between Asians and Caucasians, Maturitas. (2010) 65, no. 4, 315–319, 10.1016/j.maturitas.2009.12.012, 20079586. 20079586

[34] Lear S. A. , James P. T. , Ko G. T. , and Kumanyika S. , Appropriateness of Waist Circumference and Waist-to-Hip Ratio Cutoffs for Different Ethnic groups, European Journal of Clinical Nutrition. (2010) 64, no. 1, 42–61, 10.1038/ejcn.2009.70, 19672278. 19672278

[35] Fox C. S. , Massaro J. M. , Hoffmann U. , Pou K. M. , Maurovich-Horvat P. , Liu C. Y. , Vasan R. S. , Murabito J. M. , Meigs J. B. , Cupples L. A. , D’Agostino R. B. , and O’Donnell C. J. , Abdominal Visceral and Subcutaneous Adipose Tissue Compartments, Circulation. (2007) 116, no. 1, 39–48, 10.1161/CIRCULATIONAHA.106.675355. 17576866

[36] Reuben D. B. , Seeman T. E. , Keeler E. , Hayes R. P. , Bowman L. , Sewall A. , Hirsch S. H. , Wallace R. B. , and Guralnik J. M. , Refining the Categorization of Physical Functional Status: the Added Value of Combining Self-Reported and Performance-Based Measures, Journals of Gerontology. Series A, Biological Sciences and Medical Sciences. (2004) 59, no. 10, 1056–1061, 10.1093/gerona/59.10.M1056, 15528778. 15528778

[37] Hoogendijk E. O. , Afilalo J. , Ensrud K. E. , Kowal P. , onder G. , and Fried L. P. , Frailty: Implications for Clinical Practice and Public Health, Lancet. (2019) 394, no. 10206, 1365–1375, 10.1016/S0140-6736(19)31786-6. 31609228

[38] Hamdy O. , Porramatikul S. , and Al-Ozairi E. , Metabolic Obesity: The Paradox Between Visceral and Subcutaneous fat, Current Diabetes Reviews. (2006) 2, no. 4, 367–373, 10.2174/1573399810602040367, 18220642. 18220642

[39] Chen Y. W. , Apostolakis S. , and Lip G. Y. H. , Exercise-Induced Changes in Inflammatory Processes: Implications for Thrombogenesis in Cardiovascular Disease, Annals of Medicine. (2014) 46, no. 7, 439–455, 10.3109/07853890.2014.927713, 25012964. 25012964

[40] Hotamisligil G. S. , Inflammation and Metabolic Disorders, Nature. (2006) 444, no. 7121, 860–867, 10.1038/nature05485. 17167474

[41] McFadyen J. D. , Kiefer J. , Braig D. , Loseff-Silver J. , Potempa L. A. , Eisenhardt S. U. , and Peter K. , Dissociation of C-Reactive Protein Localizes and Amplifies Inflammation: Evidence for a Direct Biological Role of C-Reactive Protein and Its Conformational Changes, Frontiers in Immunology. (2018) 9, 10.3389/fimmu.2018.01351, 29946323. PMC6005900 29946323

[42] Ling C. , Cook M. D. , Grimm H. , Aldokhayyil M. , Gomez D. , and Brown M. , The Effect of Race and Shear Stress on CRP‐Induced Responses in Endothelial Cells, Mediators of Inflammation. (2021) 2021, no. 1, 6687250, 10.1155/2021/6687250, 34899053. 34899053 PMC8660250

[43] Donath M. Y. and Shoelson S. E. , Type 2 Diabetes as an Inflammatory disease, Nature Reviews Immunology. (2011) 11, no. 2, 98–107, 10.1038/nri2925, 21233852. 21233852

[44] Ma K. , Xia T. , Liu T. , Wang H. , Wang Q. , Ju S. , and Zhang R. , Association Between Metabolic Score for Visceral Fat and Adverse Outcomes in Chronic Obstructive Pulmonary disease, BMJ Open Respiratory Research. (2026) 13, no. 1, e003393, 10.1136/bmjresp-2025-003393, 41494698. PMC12778229 41494698

[45] Gawałko M. , Saljic A. , Li N. , Abu-Taha I. , Jespersen T. , Linz D. , Nattel S. , Heijman J. , Fender A. , and Dobrev D. , Adiposity-Associated Atrial Fibrillation: Molecular Determinants, Mechanisms, and Clinical significance, Cardiovascular Research. (2023) 119, no. 3, 614–630, 10.1093/cvr/cvac093, 35689487. 35689487 PMC10409902

[46] Bruckner F. , Gruber J. R. , Ruf A. , Edwin Thanarajah S. , Reif A. , and Matura S. , Exploring the link Between Lifestyle, Inflammation, and Insulin Resistance Through an Improved Healthy Living Index, Nutrients. (2024) 16, no. 3, 10.3390/nu16030388, 38337673. PMC10857191 38337673

[47] Spartano N. L. , Stevenson M. D. , Xanthakis V. , Larson M. G. , Andersson C. , Murabito J. M. , and Vasan R. S. , Associations of Objective Physical Activity With Insulin Sensitivity and Circulating Adipokine Profile: The Framingham Heart Study, Clinical Obesity. (2017) 7, no. 2, 59–69, 10.1111/cob.12177, 28112860. 28112860 PMC5339058

[48] Pedersen B. K. , Muscles and their Myokines, Journal of Experimental Biology. (2011) 214, no. 2, 337–346, 10.1242/jeb.048074. 21177953

[49] Cleasby M. E. , Jamieson P. M. , and Atherton P. J. , Insulin Resistance and Sarcopenia: Mechanistic Links Between Common co-morbidities, Journal of Endocrinology. (2016) 229, no. 2, R67–R81, 10.1530/JOE-15-0533, 26931135. 26931135

[50] Koenig J. , McLean K. J. , and Bishop L. , Psychological Distress and Mental Health Diagnoses in Adults by Disability and Functional Difficulty Status: Findings From the 2021 National Health Interview survey, Disability and Health Journal. (2024) 17, no. 4, 101641, 10.1016/j.dhjo.2024.101641, 38816306. 38816306 PMC11401770

[51] Wang W. , Liu Y. , Ji D. , Xie K. , Yang Y. , Zhu X. , Feng Z. , Guo H. , and Wang B. , The Association Between Functional Disability and Depressive Symptoms Among Older Adults: Findings From the China Health and Retirement Longitudinal Study (CHARLS), Journal of Affective Disorders. (2024) 351, 518–526, 10.1016/j.jad.2024.01.256, 38307133. 38307133

[52] Rosmond R. , Role of Stress in the Pathogenesis of the Metabolic syndrome, Psychoneuroendocrinology. (2005) 30, no. 1, 1–10, 10.1016/j.psyneuen.2004.05.007. 15358437

[53] Lasselin J. , Magne E. , Beau C. , and ? , Adipose Inflammation in Obesity: Relationship With Circulating Levels of Inflammatory Markers and Association With Surgery-Induced Weight Loss, Journal of Clinical Endocrinology and Metabolism. (2014) 99, no. 1, E53–E61, 10.1210/jc.2013-2673, 24243638. 24243638

[54] Gregor M. F. and Hotamisligil G. S. , Inflammatory Mechanisms in Obesity, Annual Review of Immunology. (2011) 29, no. 1, 415–445, 10.1146/annurev-immunol-031210-101322. 21219177

[55] Dantzer R. , O′connor J. C. , Freund G. G. , Johnson R. W. , and Kelley K. W. , From Inflammation to Sickness and Depression: When the Immune System Subjugates the brain, Nature Reviews Neuroscience. (2008) 9, no. 1, 46–56, 10.1038/nrn2297, 18073775. 18073775 PMC2919277

[56] Nair K. S. , Aging Muscle, American Journal of Clinical Nutrition. (2005) 81, no. 5, 953–963, 10.1093/ajcn/81.5.953. 15883415

[57] Chien S. C. , Chiu H. C. , Chiu Y. C. , and ? , Clinical Relevancies of Sarcopenic Obesity in Patients With Metabolic Dysfunction-Associated Fatty Liver Disease (MASLD), Digestive Diseases and Sciences. (2025) 70, no. 3, 1190–1200, 10.1007/s10620-025-08844-z, 39826065. 39826065

[58] Kim T. N. , Park M. S. , Lim K. I. , Choi H. Y. , Yang S. J. , Yoo H. J. , Kang H. J. , Song W. , Choi H. , Baik S. H. , Choi D. S. , and Choi K. M. , Relationships Between Sarcopenic Obesity and Insulin Resistance, Inflammation, and vitaminDstatus: theKoreanSarcopenicObesityStudy, Clinical Endocrinology. (2013) 78, no. 4, 525–532, 10.1111/j.1365-2265.2012.04433.x, 22563924. 22563924

[59] Zhang H. , Duan X. , Rong P. , Dang Y. , Yan M. , Zhao Y. , Chen F. , Zhou J. , Chen Y. , Wang D. , and Pei L. , Effects of Potential Risk Factors on the Development of Cardiometabolic Multimorbidity and Mortality Among the Elders in China, Frontiers in Cardiovascular Medicine. (2022) 9, 966217, 10.3389/fcvm.2022.966217, 36158847. 36158847 PMC9502033

[60] El Khoudary S. R. , Aggarwal B. , Beckie T. M. , Hodis H. N. , Johnson A. E. , Langer R. D. , Limacher M. C. , Manson J. E. , Stefanick M. L. , Allison M. A. , American Heart Association Prevention Science Committee of the Council on Epidemiology and Prevention , and Council on Cardiovascular and Stroke Nursing , Menopause Transition and Cardiovascular Disease Risk: Implications for Timing of Early Prevention: A Scientific Statement From the American Heart Association, Circulation. (2020) 142, no. 25, e506–e532, 10.1161/CIR.0000000000000912, 33251828. 33251828

[61] Hu Y. , Zong G. , Liu G. , Wang M. , Rosner B. , Pan A. , Willett W. C. , Manson J. A. E. , Hu F. B. , and Sun Q. , Smoking Cessation, Weight Change, Type 2 Diabetes, and Mortality, New England Journal of Medicine. (2018) 379, no. 7, 623–632, 10.1056/NEJMoa1803626, 30110591. 30110591 PMC6165582

[62] Martinez-Urbistondo D. , Perez-Diaz-del-Campo N. , Landecho M. F. , and Martinez J. A. , Alcohol Drinking Impacts on Adiposity and Steatotic Liver Disease: Concurrent Effects on Metabolic Pathways and Cardiovascular Risks, Current Obesity Reports. (2024) 13, no. 3, 461–474, 10.1007/s13679-024-00560-5, 38520634. 38520634 PMC11306502

[63] Tang Y. , Lin D. , Xu H. , Xu L. , Guo S. , Zheng X. , Su M. , Zeng K. , Feng W. , Ye J. , and Wang L. , Global Burden and Projections of Cardiometabolic Diseases Attributable to High Alcohol Use: A Comparative Risk Assessment Based on the GBD 2021 study, Frontiers in Nutrition. (2026) 13, 1698730, 10.3389/fnut.2026.1698730, 41867675. 41867675 PMC13002442

[64] Jurj A. L. , Wen W. , Li H. L. , Zheng W. , Yang G. , Xiang Y. B. , Gao Y. T. , and Shu X. O. , Spousal Correlations for Lifestyle Factors and Selected Diseases in Chinese Couples, Annals of Epidemiology. (2006) 16, no. 4, 285–291, 10.1016/j.annepidem.2005.07.060, 16257231. 16257231

[65] Pollak C. , Verghese J. , Buchman A. S. , Jin Y. , and Blumen H. M. , Loneliness Predicts Progression of Frailty in Married and Widowed, but Not Unmarried Community Dwelling Older Adults, Journal of Frailty & Aging. (2024) 13, no. 2, 163–171, 10.14283/jfa.2024.27, 38616373. 38616373 PMC11898203

[66] Wieczorek M. , Meier C. , Vilpert S. , Reinecke R. , Borrat-Besson C. , Maurer J. , and Kliegel M. , Association Between Multiple Chronic Conditions and Insufficient Health Literacy: Cross-Sectional Evidence From a Population-Based Sample of Older Adults Living in Switzerland, BMC Public Health. (2023) 23, no. 1, 10.1186/s12889-023-15136-6, 36747134. PMC9901105 36747134

[67] Neeland I. J. , Ross R. , Després J. P. , Matsuzawa Y. , Yamashita S. , Shai I. , Seidell J. , Magni P. , Santos R. D. , Arsenault B. , Cuevas A. , Hu F. B. , Griffin B. , Zambon A. , Barter P. , Fruchart J. C. , Eckel R. H. , International Atherosclerosis Society , and International Chair on Cardiometabolic Risk Working Group on Visceral Obesity , Visceral and Ectopic Fat, Atherosclerosis, and Cardiometabolic Disease: A Position statement, Lancet Diabetes & Endocrinology. (2019) 7, no. 9, 715–725, 10.1016/S2213-8587(19)30084-1, 31301983. 31301983

[68] Dent E. , Martin F. C. , Bergman H. , Woo J. , Romero-Ortuno R. , and Walston J. D. , Management of Frailty: Opportunities, Challenges, and Future directions, Lancet. (2019) 394, no. 10206, 1376–1386, 10.1016/S0140-6736(19)31785-4, 31609229. 31609229

