# Supplementary material for: Exploring the Joint Impact of METS‐VF and Functional Limitation on Cardiometabolic Multimorbidity Risk
Source: J Diabetes Res. 2026 Jun 12;2026:6450867. doi: 10.1155/jdr/6450867 (PMC13261289; doi:10.1155/jdr/6450867)
Supplement: Supplementary file 1 — Supporting Information Additional supporting information can be found online in the Supporting Information section. Table S1 Sensitivity analyses evaluating the robustness of the associations between METS‐VF, functional limitation, and incident CMM. [file JDR-2026-6450867-s001.docx]

| **Variable and Category** | **Panel A: Excluding Baseline Medication Users** | **Panel B: Lag Analysis (Excluding Cases within First 2 Years)** | **Panel C: Super-Clean Cohort (Excluding Any Single Baseline CMM Component)** |
| --- | --- | --- | --- |
|  | HR (95% CI) ; P value | HR (95% CI) ; P value | HR (95% CI) ; P value |
| METS-VF |  |  |  |
| Continuous (per 1 unit) | 1.16 (1.10 - 1.22) ; <0.001 | 1.18 (1.12 - 1.23) ; <0.001 | 1.14 (1.07 - 1.22) ; <0.001 |
| METS-VF Group |  |  |  |
| Low (< Inflection) | 1.00 (Reference) | 1.00 (Reference) | 1.00 (Reference) |
| High (≥ Inflection) | 1.78 (1.34 - 2.35) ; <0.001 | 1.64 (1.27 - 2.12) ; <0.001 | 1.67 (1.10 - 2.51) ; 0.015 |
| Functional Limitation |  |  |  |
| Continuous (per 1 unit) | 1.13 (1.08 - 1.18) ; <0.001 | 1.11 (1.07 - 1.15) ; <0.001 | 1.16 (1.08 - 1.25) ; <0.001 |
| Joint Effect Category |  |  |  |
| 1. Neither high | 1.00 (Reference) | 1.00 (Reference) | 1.00 (Reference) |
| 2. High Func_limt only | 1.36 (0.79 - 2.33) ; 0.267 | 1.32 (0.81 - 2.16) ; 0.270 | 1.18 (0.54 - 2.59) ; 0.680 |
| 3. High METS-VF only | 1.70 (1.19 - 2.43) ; 0.004 | 1.53 (1.11 - 2.11) ; 0.010 | 1.38 (0.82 - 2.32) ; 0.220 |
| 4. Both high | 2.49 (1.72 - 3.61) ; <0.001 | 2.33 (1.67 - 3.25) ; <0.001 | 2.53 (1.47 - 4.34) ; <0.001 |

Table S1 Sensitivity analyses evaluating the robustness of the associations between METS-VF, functional limitation, and incident cardiometabolic multimorbidity.
